# The influence of social withdrawal and depression on the self-esteem of female adolescents: The mediating effect of grit

**DOI:** 10.1371/journal.pone.0288530

**Published:** 2023-07-12

**Authors:** Donguk Lee, Sangmi Lee

**Affiliations:** 1 Department of Educational Administration, The Graduate School of Government and Business, Yonsei University, Wonju-si, Gangwon-do, Republic of Korea; 2 College of Nursing, Dongyang University, Yeongju-si, Gyeongsangbuk-do, Republic of Korea; National University of Sciences and Technology, PAKISTAN

## Abstract

Previous studies have shown that female adolescents are at a higher risk of low self-esteem than male adolescents, and self-esteem in adolescents is critical for academic performance, adult health, and economic status. Depression, social withdrawal, and grit are predicted to be internal factors that affect self-esteem, and an integrated exploration of the relationship between them is required for a proper approach to enhance self-esteem in female adolescents. Therefore, this study investigated the influence of social withdrawal and depression on self-esteem among female adolescents and explored the mediating effect of grit on self-esteem. Data collected from 1,106 girls in the third year of middle school of the third-year survey (2020) of the Korean Children and Youth Panel Survey 2018 were analyzed in this study. For data analysis, partial least square-structural equation modeling was performed using SmartPLS 3.0. Social withdrawal was negatively related to grit, but not related to self-esteem. Depression was negatively related to grit and self-esteem. Grit was positively related to self-esteem. In addition, grit showed mediating effects in the associations between social withdrawal and self-esteem, and between depression and self-esteem in female adolescents. In conclusion, in female adolescents, the mediating effects of grit attenuated the negative effects of social withdrawal and depression on self-esteem. To enhance self-esteem in female adolescents, it is important to develop and implement strategies to strengthen grit and control negative emotional states, such as depression.

## Introduction

Self-esteem is a subjective evaluation of the self, referring to how valuable one feels [1]. It is necessary for adolescents to develop good self-esteem because they are likely to experience emotional instability amid psychological confusion and internal conflicts caused by rapid physical changes. Low self-esteem in adolescents is associated with problem behaviors such as delinquency and suicide attempts [2, 3], poor academic achievement [4], negative emotions such as depression and anxiety [5–7], poor health, criminal behavior, and limited economic prospects in adulthood [8]. Therefore, adolescents’ self-esteem is an important factor with long-term effects on later life. Studies have consistently reported that female adolescents have lower self-esteem than male adolescents [9–12]; thus, it is necessary to explore ways to improve female adolescents’ self-esteem based on an assessment of factors that affect self-esteem in this population.

Female adolescents are more focused on their emotional states than male adolescents, but have difficulty recognizing their emotions clearly and a limited ability to recover from negative emotional states [9]. For this reason, female adolescents are more vulnerable to negative emotional states than male adolescents. Negative emotional states are related to low self-esteem [9, 13, 14]. A previous study found that depression and anxiety, as mental health factors, had greater effects on adolescents’ self-esteem than family affluence and personality [15]. Hence, the factors influencing self-esteem in female adolescents need to be explored from the emotional standpoint.

Depression is a common emotional problem among Korean adolescents. An analysis of treatment by the Health Insurance Review & Assessment Service [16] showed that the number of teenage depression patients in Korea increased from 30,273 in 2017 to 57,587 in 2021, corresponding to a 90.2% increase over 5 years, and the annual average incidence rate is 17.4% of all adolescents. This is much higher than the depression rates of 1.1% in 10- to 14-year-olds and 2.8% in 15- to 19-year-olds estimated by the World Health Organization [17]. Since suicide is the top cause of death among adolescents in Korea [18], the high incidence of depression in adolescents is a very worrisome social problem in Korea. Since the incidence of depression among women is about twice that of men [16], and depression is related to low self-esteem [13, 14], the effect of depression on self-esteem in Korean adolescent girls warrants further research.

Social withdrawal refers to a state of social isolation involving the intentional avoidance of interactions in social relationships or contact with others, and difficulty in properly participating in social situations [19]. In adolescents, social withdrawal manifests as refusal to attend school and to contact family or friends [20]; thus, social withdrawal is a major threat to the successful growth of adolescents as members of society. In addition, social withdrawal exacerbates emotional problems such as depression, isolation, and anxiety in the long term, and leads to a negative perception of oneself [19]. Therefore, it would be reasonable to predict that social withdrawal during adolescence has a negative effect on self-esteem. However, previous studies have mainly investigated self-esteem as an important factor influencing the emotional state of adolescents [5–7], whereas relatively few studies have focused on factors affecting self-esteem in adolescents, especially negative emotional states, such as depression or social withdrawal. Social withdrawal, like depression, has been reported to be more common in female adolescents than in male adolescents [19]. Thus, it is necessary to explore in greater depth the problems caused by emotional states in female adolescents.

Grit, which refers to the consistency of interest, passion and persistence to achieve long-term goals [21], is an important positive psychological characteristic related to an individual’s goal achievement, life satisfaction, and positive emotions [21]. A previous study showed that grit significantly affected self-esteem in male and female adolescents [22]. Other studies have suggested that negative emotional states (e.g., depression or social withdrawal) in adolescents have a high likelihood of weakening grit [23, 24], but related knowledge is still incomplete. Grit also shows differences according to gender, and some previous studies have reported that female adolescents have lower levels of grit than male adolescents [25]. Therefore, the effects of depression and social withdrawal on grit in female adolescents should be studied.

Emotional problems that have recently emerged among Korean adolescents are likely to have a negative impact on self-esteem, and female adolescents are particularly at risk of developing low self-esteem. Therefore, it is essential to investigate how the emotional problems of adolescent girls affect self-esteem, but related studies are still lacking. Furthermore, examining the mediating role of grit in the relationship between depression, social withdrawal and self-esteem, will present a new perspective that will help derive measures to improve self-esteem in adolescent girls.

Socio-economic status is also known to be significantly associated with the development of self-esteem, especially during the significant stage of adolescence. For instance, adolescents with lower family income were found to have lower self-esteem than those with higher family income [26]. Therefore, family economic status (FES) is a meaningful factor that should be considered when exploring the relationship between self-esteem and related variables in adolescents.

### Social withdrawal → grit and social withdrawal → self-esteem

To the best of the authors’ knowledge, no studies have explored the relationship between social withdrawal and grit in adolescents. However, support from social relationships with adults, such as parents, teachers, and mentors, is important for improving grit [21], and peer attachment was found to have a positive effect on grit in adolescents [27]. Therefore, adolescents who avoid social relationships and feel a sense of withdrawal from relationships with others are more likely to have low levels of grit.

According to previous studies on the relationship between social withdrawal and self-esteem, shyness (a sub-concept of social withdrawal) had a negative effect on self-esteem [28, 29], and social phobia strongly influenced self-esteem in adolescence [12]. Adaptation to school, which is the most immediate social environment for middle-school and high-school adolescents, was found to affect self-esteem [14]. In addition, a study on college students found that shyness and avoidance, which are symptoms of social withdrawal, showed negative correlations with self-worth, and shyness was identified as a negative predictor of self-esteem [29]. That is, a greater degree of social withdrawal was associated with a higher risk of lowered self-esteem in adolescence.

### Depression → grit, and depression → self-esteem

Previous research has found that emotional problems, such as depression and anxiety, were inversely related to grit in college students [13]. In addition, grit was found to negatively affect depression [23], and high level of grit in adolescents with school burnout significantly reduced depressive symptoms [30].

Studies on the relationship between depression and self-esteem have shown that depression and anxiety in adolescents were the factors with the strongest effect on self-esteem [15], and depression in college students was negatively related to self-esteem [13]. Furthermore, among variables such as depression, social support, body image, problem behavior, and school life adjustment, depression had the greatest effect on self-esteem in middle- and high-school students [14]. Adolescents’ mental well-being and self-esteem were shown to be mutually influencing, and mental health status, a concept similar to depression, had an effect on self-esteem [11].

### Grit → self-esteem

Previous studies on the relationship between grit and self-esteem have shown that grit had a significant effect on self-esteem and other concepts related to self-cognition such as self-efficacy, self-control, self-consciousness, self-satisfaction, and self-awareness [23, 31–34].

### The mediating role of grit

A meta-analysis confirmed the negative correlation between grit and negative emotional outcomes such as depression [35], and a previous study implied the possible buffering role of grit in depression caused by negative personality traits [13]. Grit has also been reported to play a partial mediating role in the relationship between depression and self-resilience, which is a concept similar to self-esteem [36]. Grit also played a mediating role in the negative relationship between self-esteem and social withdrawal in elementary-school students [37].

### Research hypotheses and model

**Fig 1:**
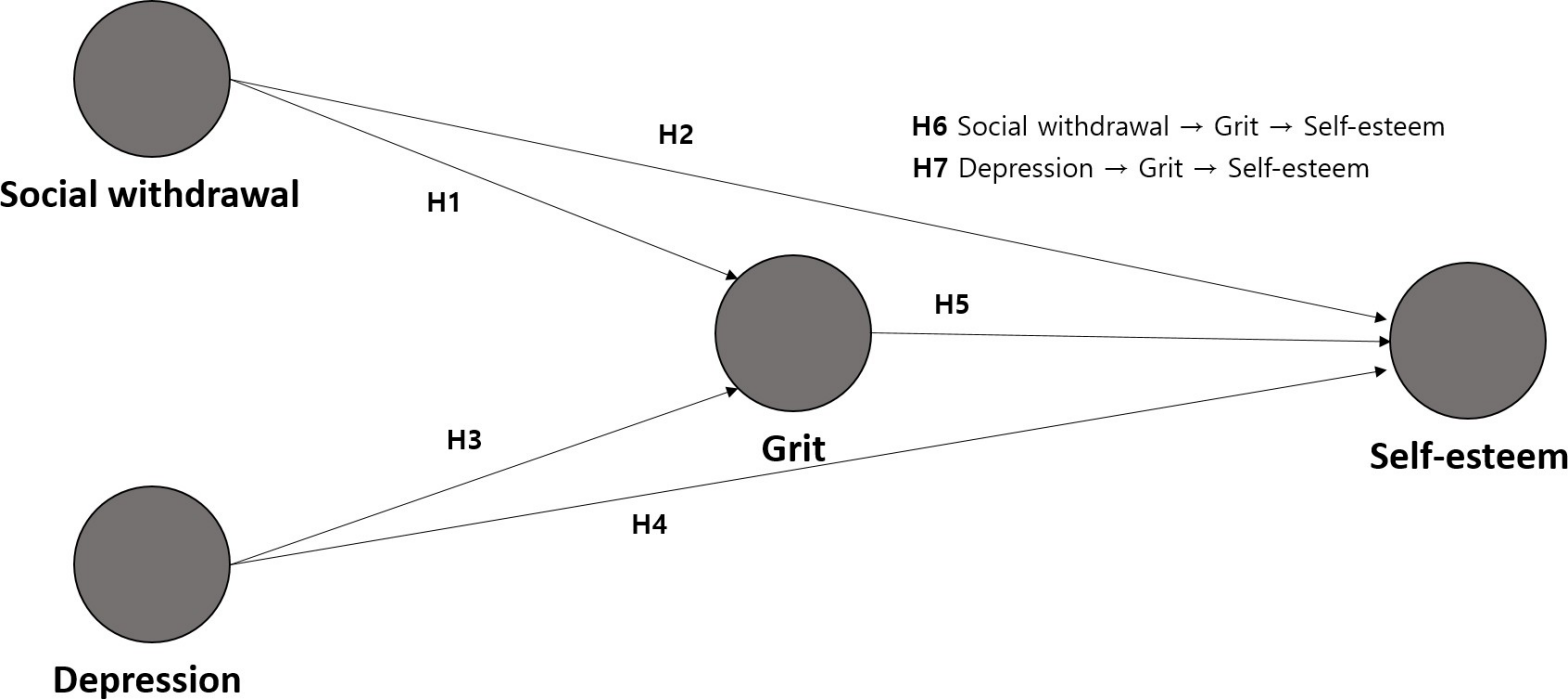
Research model.

In this study, research hypotheses and a model were established based on previous studies to clarify the structural relationships of social withdrawal, depression, grit, and self-esteem, as follows (Fig 1).

Hypothesis 1 (H1). Social withdrawal significantly affects grit.

Hypothesis 2 (H2). Social withdrawal significantly affects self-esteem.

Hypothesis 3 (H3). Depression significantly affects grit.

Hypothesis 4 (H4). Depression significantly affects self-esteem.

Hypothesis 5 (H5). Grit significantly affects self-esteem.

Hypothesis 6 (H6). Social withdrawal significantly affects self-esteem by mediating grit.

Hypothesis 7 (H7). Depression significantly affects self-esteem by mediating grit.

### Study purpose

The purpose of this study was to investigate the effects of social withdrawal, depression, and grit on self-esteem among Korean adolescent girls. The specific aims were 1) to investigate the effects of social withdrawal, depression, and grit on self-esteem; 2) to examine whether grit mediates the effects of social withdrawal and depression on self-esteem in female adolescents; and 3) to identify FES-related differences in the relationship between social withdrawal, depression, and grit on self-esteem in female adolescents.

## Methods

### Study design

This study was a secondary analysis of panel data from the Korean Children and Youth Panel Survey 2018 (KCYPS 2018) to characterize the relationships among social withdrawal, depression, grit, and self-esteem in female adolescents by structural equation modeling (SEM).

### Subjects and data collection

The subjects in this study were Korean adolescent girls, who comprised the first middle school cohort panel of the KCYPS 2018 and were in the third year of middle school. The original panel of the KCYPS 2018 contained 2,590 participants from schools extracted by probability proportional to size sampling among all middle schools nationwide in Korea. In this study, considering that emotional problems increase rapidly in mid-adolescence [11], only data from the third year (2020) were used from the three-year data of the KCYPS 2018. Thus, among the 2,384 participants in the third year (2020), a total of 1,106 female adolescents were selected as final subjects in this study.

The KCYPS 2018 contains panel data collected by the National Youth Policy Institute in order to construct data that can be used to analyze systematically and multi-dimensionally the complex changes related to the growth and development of children and adolescents in Korea. These data have been collected through follow-up surveys every year, starting from 2018, through individual interviews targeting the original panel of first-year middle school students, and data for 2020 were collected through online surveys when individual interviews were not possible due to the impact of COVID-19.

### Ethical considerations

The current study was conducted with an approval of exemption from ethical review from the Institutional Review Board of the institution to which the researcher is affiliated (IRB No.: 1041495-202208-HR-01-01), because it was a study using secondary data based on publicly accessible and non-identifiable data provided from the third survey of the KCYPS 2018 of the National Youth Policy Institute in Korea (https://www.nypi.re.kr/archive/mps).

### Measurements

To investigate the relationships among the variables of social withdrawal, depression, and grit affecting the self-esteem of Korean adolescent girls, the tools utilized to survey participants in the KCYPS 2018 were used, and the detailed variables were defined and classified according to the KCYPS 2018 User Guide [38]. The dependent variable of the study was self-esteem, the exogenous variables were social withdrawal and depression, and the mediating variable was grit. FES was also included as a control variable in this study, and it was defined based on the economic status of the family reported by the adolescents’ parents. The responses were scored on a 5-point Likert scale ranging from 1 point for “lowest” to 5 points for “highest,” and a higher score indicated a higher family economic level.

#### Social withdrawal

Social withdrawal was evaluated using Kim and Kim’s [39] behavioral problem scale for children and adolescents, composed of 5 items, including interpersonal relationships, shame, confidence in expressing opinions, modesty, and timidity (e.g., “’I find it difficult to express my opinions clearly to others” and “I do not like to be in front of people”). The responses were scored on a 4-point Likert scale ranging from 1 point for “not at all” to 4 points for “strongly agree,” with a higher score indicating higher social withdrawal. Each item in this study was used as a measurement variable, and social withdrawal was set as a latent variable and reflected in the research model. In this study, Cronbach’s α of this tool was .88.

#### Depression

Depression was measured using depression scale, which consisted of 10 items (e.g., “I feel unhappy or sad and depressed” and “’I have no interest in anything”), developed by Kim et al [40]. There were 3 to 4 items for each three sub-domains (worry or anxiety, loss of motivation, and negative thoughts). The responses were scored on a 4-point Likert scale ranging from 1 point for “not at all” to 4 points for “strongly agree,” and a higher score indicated a higher degree of depression. In this study, Cronbach’s α of this tool was .92.

#### Grit

Grit was evaluated using Kim and Hwang’s [41] Korean GRIT scale for children, which consisted of 8 items (e.g., “I am not very frustrated when difficulties arise while solving problems,” “I stop being frustrated faster than others,” and “I am diligent”). There were 2 to 3 items for each three sub-domains (passion or diligence, interest or perseverance, and concentration of effort). The responses were scored on a 4-point Likert scale, ranging from 1 point for “not at all” to 4 points for “strongly agree,” with a higher score indicating higher grit. The scores for four inverse items (e.g., “If it takes a long time to complete something, it is difficult for me to keep working hard”) were calculated by inverse conversion, and Cronbach’s α in this study was .75.

#### Self-esteem

Self-esteem was measured using Rosenberg’s self-esteem scale [42], translated by National Youth Policy Institute. This scale was composed of 10 items (e.g., “I feel that I am at least as valuable as others” and “I have a positive attitude toward myself”). There were 3 to 4 items for each of the three sub-domains (self-respect or satisfaction, positive attitude or confidence, and advantage or value). The responses were scored on a 4-point Likert scale, ranging from 1 point for “not at all” to 4 points for “strongly agree,” and a higher score indicated higher self-esteem. The scores for four inverse items (e.g., “Sometimes I feel useless”) were calculated by inverse conversion, and Cronbach’s α in this study was .85.

### Data analysis

The absolute values of skewness and kurtosis of the main variables of the study were 0.00 to 0.68 and 0.02 to 0.95, respectively, and the data were analyzed after confirming that the normality criteria (skewness: absolute value less than 2, kurtosis: absolute value less than 7) were satisfied [43]. The subjects’ general characteristics and major variables were described with descriptive statistics (frequency, percentage, mean, and standard deviation), and the relationships between variables were examined by Pearson correlation analysis using SPSS 28.0 (IBM Corp., Armonk, NY, USA). The structural relationships among social withdrawal, depression, grit, and self-esteem were analyzed using SmartPLS 3.3.7 (SmartPLS GmbH, Germany) through partial least squares (PLS)-based SEM analysis.

To evaluate the overall measurement model, Cronbach’s α, rho_A, composite reliability (CR), average variance extracted (AVE), outer loading relevance, and indicator reliability were checked to confirm internal consistency reliability, convergent validity, and discriminant validity. To verify the fit of the structural model, χ^2^, the standardized root mean square residual (SRMR), and the normed fit index (NFI) were calculated, and the internal variance inflation factor, R^2^, f^2^, and Q^2^ were checked to evaluate the structural model. To verify the hypothesis of the structural model, the path coefficient, standard deviation, t-value, p-value, and BCa 95% CI were calculated to identify direct and indirect effects.

For the analysis and hypothesis verification regarding the role of FES in the relationship of social withdrawal, depression, and grit with self-esteem among female adolescents, the participants were dichotomized into low FES (responses of “low” and “lowest”) and high FES (responses of “average,” high,” and “highest”).

## Results

### Descriptive statistics and correlation analysis

**Fig 2:**
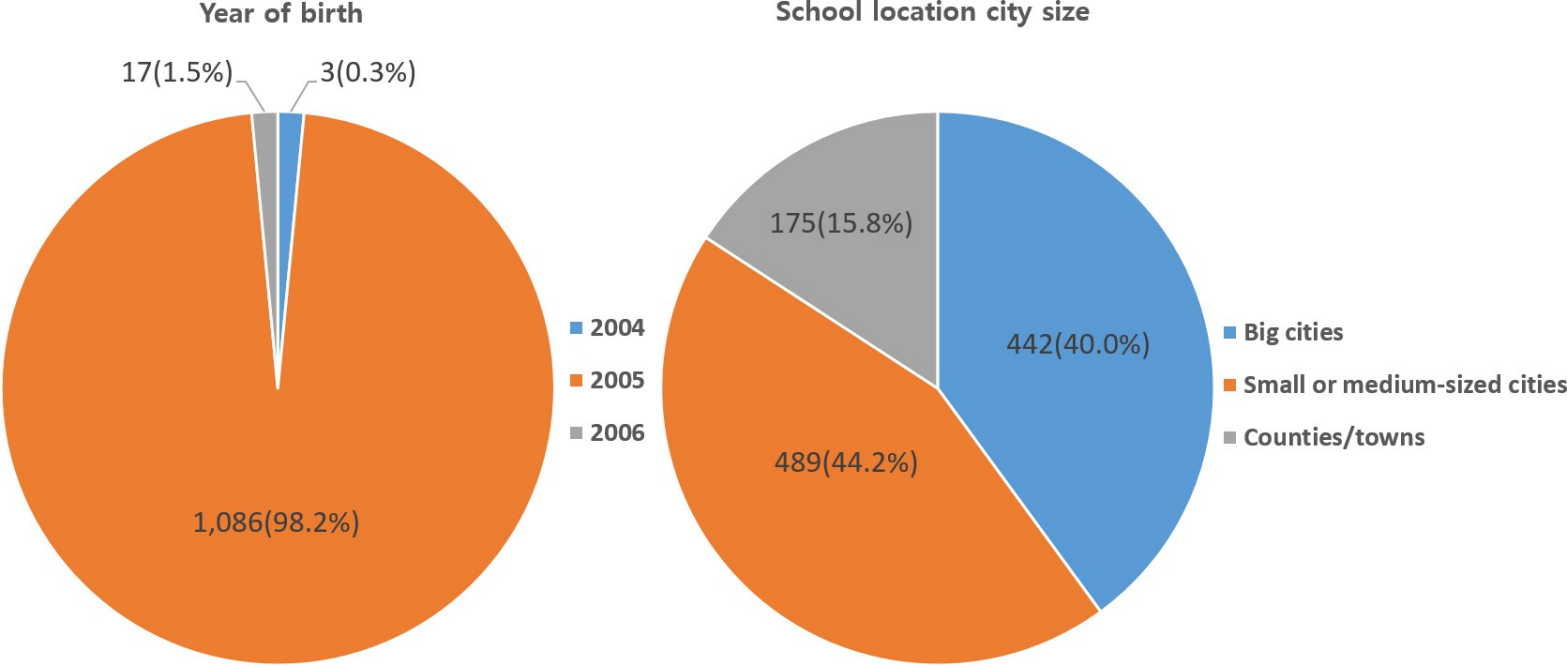
General characteristics of participants (N = 1,106).

Most of the subjects of this study were born in 2005 (1,086 persons, 98.2%). In total, 489 students (44.2%) attended schools in small or medium cities, 442 students (40.0%) in large cities, and 175 students (15.8%) in villages (Fig 2). The mean scores for social withdrawal, depression, grit, and self-esteem, which were the main variables of this study, were 2.07 (±0.85) to 2.33 (±0.85), 1.76 (±0.64) to 1.90 (±0.68), 2.51 (±0.50) to 2.62 (±0.54), and 2.73 (±0.48) to 3.01 (±0.51), respectively.

A positive correlation was found between social withdrawal and depression, while social withdrawal showed negative correlations with grit and self-esteem. Depression was negatively correlated with grit and self-esteem, and grit was positively correlated with self-esteem (Table 1).

**Table 1:** Descriptive statistics and correlation analysis of major variables.

Correlation		Social withdrawal					Depression			Grit			Self-esteem		
		➀	➁	➂	➃	➄	➀	➁	➂	➀	➁	➂	➀	➁	➂
**Social withdrawal**	**➀**														
	**➁**	.61**													
	**➂**	.57**	.64**												
	**➃**	.49**	.71**	.58**											
	**➄**	.53**	.57**	.60**	.58**										
**Depression**	**➀**	.45**	.46**	.49**	.41**	.39**									
	**➁**	.41**	.46**	.49**	.39**	.32**	.80**								
	**➂**	.45**	.40**	.50**	.38**	.39**	.81**	.75**							
**Grit**	**➀**	-.22**	-.23**	-.26**	-.22**	-.21**	-.42**	-.37**	-.35**						
	**➁**	-.23**	-.23**	-.25**	-.22**	-.22**	-.31**	-.29**	-.27**	.53**					
	**➂**	-.26**	-.23**	-.25**	-.19**	-.25**	-.35**	-.32**	-.31**	.50**	.48**				
**Self-esteem**	**➀**	-.32**	-.32**	-.38**	-.29**	-.25**	-.59**	-.56**	-.56**	.42**	.33**	.30**			
	**➁**	-.28**	-.25**	-.33**	-.25**	-.23**	-.55**	-.50**	-.52**	.44**	.26**	.37**	.64**		
	**➂**	-.25**	-.21**	-.26**	-.25**	-.22**	-.48**	-.42**	-.46**	.40**	.25**	.28**	.63**	.68**	
**Mean**		2.09	2.27	2.07	2.33	2.30	1.90	1.89	1.76	2.51	2.52	2.62	2.73	3.01	2.93
**SD**		0.92	0.90	0.85	0.85	0.91	0.68	0.71	0.64	0.50	0.52	0.54	0.48	0.51	0.56

**p < .01 / ➀, ➁, ➂, ➃, ➄ (Observed variables for each latent variable)

### Evaluation of overall measurement model

We checked the SEM fit, following Shin’s [44] recommendation to evaluate the Cronbach’s α, CR, AVE, outer loading value, t-value, R^2^, f^2^, and Q^2^, because PLS-SEM using SmartPLS does not provide evaluation indicators for the overall goodness-of-fit (e.g., GFI and AGFI), unlike CB-SEM in AMOS.

Internal consistency reliability, convergent validity (Table 2), and discriminant validity (Table 3) were checked to evaluate the reflective measurement model of this study. Cronbach’s α was 0.75 to 0.92 (reference value 0.7 or more), rho_A was .77 to .92 (reference value .7 or more), and CR was .86 to .95 (reference value 0.7 or higher). All test values of internal consistency reliability met the standard values, confirming that the internal consistency reliability was satisfactory [45–48].

**Table 2:** Internal consistency reliability and convergent validity.

Latent variables		Outer loading	Indicator reliability	Cronbach’s α	rho_A	CR	AVE
**Social withdrawal**	**Interpersonal relationship**	0.79	0.62	0.88	0.88	0.91	0.67
	**Shame**	0.86	0.74				
	**Confidence in expressing opinions**	0.84	0.71				
	**Modest**	0.81	0.66				
	**Timid**	0.79	0.63				
**Depression**	**Worry or anxiety**	0.94	0.89	0.92	0.92	0.95	0.86
	**Loss of motivation**	0.91	0.84				
	**Negative thoughts**	0.92	0.85				
**Grit**	**Passion of diligence**	0.86	0.74	0.75	0.77	0.86	0.67
	**Interest or perseverance**	0.79	0.62				
	**Concentration or effort**	0.80	0.64				
**Self-esteem**	**Respect or satisfaction**	0.88	0.77	0.85	0.85	0.91	0.77
	**Positive attitude or confidence**	0.89	0.78				
	**Advantage or value**	0.86	0.75				

**Table 3:** Discriminant validity in the measurement model.

Latent variable	Social withdrawal	Depression	Grit	Self-esteem
**Social withdrawal**	0.82			
**Depression**	0.57	0.92		
**Grit**	-0.34	-0.44	0.82	
**Self-esteem**	-0.39	-0.64	0.48	0.87

To check whether convergent validity was met, the outer loading relevance was 0.79 to 0.94 (reference value 0.7 or more), indicator reliability was 0.62 to 0.89 (reference value 0.5 or more), and the AVE was 0.67 to 0.86 (reference value of 0.5 or more); thus, convergent validity was satisfactory [49–52].

The discriminant validity of this study model was satisfactory, as the square root value of the AVE was uniformly higher than the correlation values between the latent variables [52].

### Structural model results and hypothesis verification

**Fig 3:**
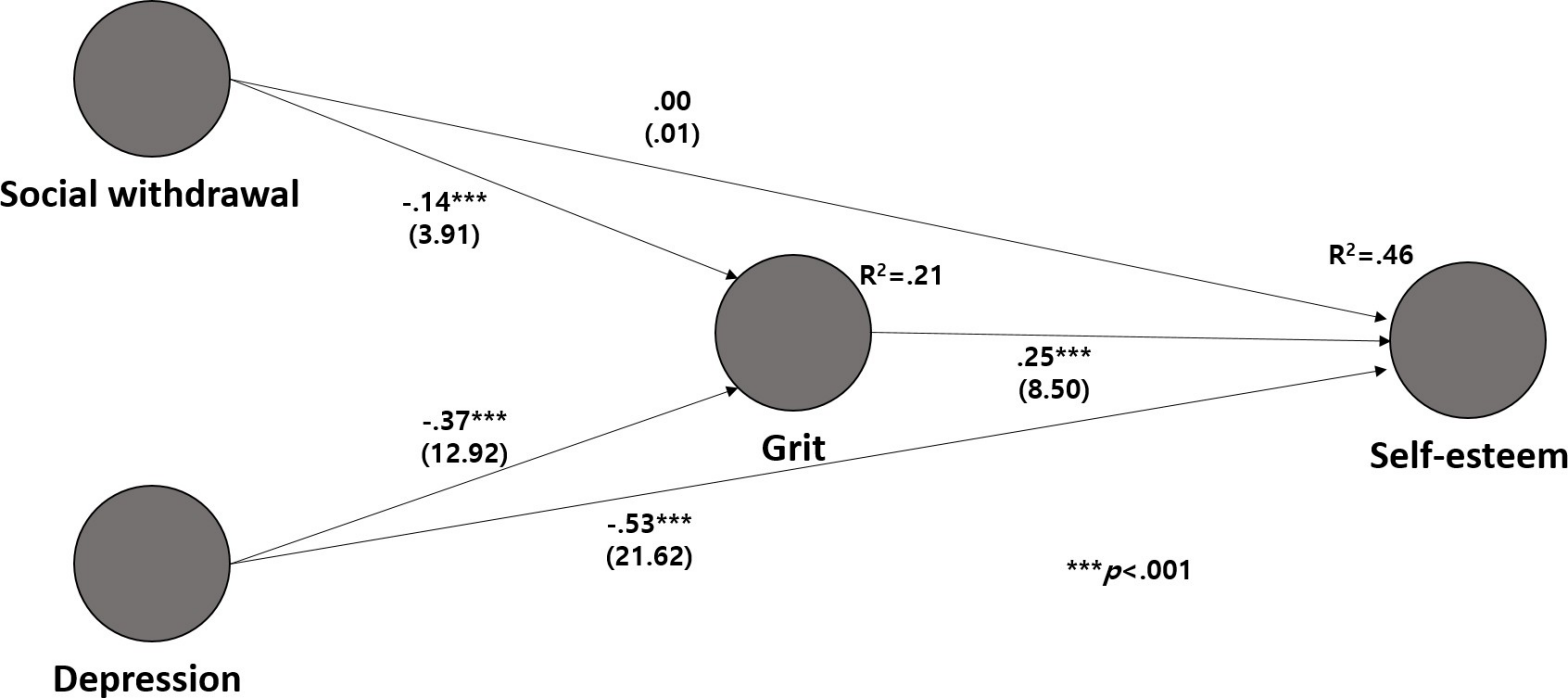
Results of hypothesis testing for the structural model.

To test the model fit, χ^2^, SRMR, and NFI were checked. The goodness of fit of the model was confirmed (χ^2^ = 1162.203, SRMR = 0.057 [reference value, less than 0.08], and NFI = 0.870 [reference value, good fit when close to 1]) [53, 54]. Because SmartPLS does not allow correlations (via double-headed arrows) between variables in the model [55], correlations between latent variables were not expressed in the SEM model (Fig 3).

Since the internal variance inflation factor of the structural model in this study ranged from 1.27 to 1.64, which is less than the reference value of 5, there was no problem of multicollinearity between latent variables [56], and the modified R^2^ values of grit and self-esteem were 0.21 and 0.46, respectively. Self-esteem was the endogenous variable with the largest explanatory power among the latent variables, with an explanatory power ranging between weak and medium [50].

The effect sizes (f^2^) of social withdrawal and depression on grit were 0.016 and 0.116, respectively, and the effect sizes (f^2^) of social withdrawal and depression on self-esteem were 0.000 and 0.319, respectively. Depression had the largest effect size (f^2^) on self-esteem, and it can be interpreted as between medium and large [51, 57].

The Q^2^ values were 0.14 for grit and 0.35 for self-esteem; these values were greater than 0, confirming that the structural model of this study secured predictive suitability [58–60].

Hypothesis testing on the direct effects of the structural model confirmed that social withdrawal (H1: β = -0.14, *p* < .001) and depression (H3: β = -0.37, *p* < .001) had significant negative effects on grit. Depression had a significant negative effect on self-esteem (H4: β = -0.53, *p* < .001), while social withdrawal had no significant effect (H2). Grit had a significant positive effect on self-esteem (H5: β = 0.25, *p* < .001) (Fig 3) (Table 4).

**Table 4:** Research hypothesis testing.

Hypothesized path		Path coefficient	Stdev	t (*p*)	BCa 95% CI	Decision
**Direct effects**						
**H1**	**Social withdrawal → Grit**	-0.14	0.03	3.91 (< .001)	[-0.21, -0.07]	Supported
**H2**	**Social withdrawal → Self-esteem**	0.00	0.03	0.01 (.995)	[-0.05, 0.05]	Not supported
**H3**	**Depression → Grit**	-0.37	0.03	12.92 (< .001)	[-0.42, -0.32]	Supported
**H4**	**Depression → Self-esteem**	-0.53	0.02	21.62 (< .001)	[-0.58, -0.49]	Supported
**H5**	**Grit → Self-esteem**	0.25	0.03	8.50 (< .001)	[0.19, 0.30]	Supported
**Indirect effects**						
**H6**	**Social withdrawal → Grit → Self-esteem**	-0.03	0.01	3.38 (< .001)	[-0.06, -0.02]	Supported
**H7**	**Depression → Grit → Self-esteem**	-0.09	0.01	6.87 (< .001)	[-0.12, -0.07]	Supported

Hypothesis testing of the mediating effect in the structural model showed that social withdrawal (H6: β = -0.03, *p* < .001) and depression (H7: β = -0.09, *p* < .001) had significant negative influences on self-esteem through grit (Table 4).

### Research hypothesis testing according to FES groups

Social withdrawal (H1: β = -0.23, *p* = .005) in female adolescents from the low-FES group had a significant negative effect on grit, but no effect on self-esteem(H2). Depression had negative effects on grit (H3: β = -0.49, *p* < .001) and self-esteem (H4: β = -0.59, *p* <. 001). Grit had a positive effect on self-esteem (H5: β = 0.22, *p* = .002). Social withdrawal had a negative effect on self-esteem (H6: β = -0.05, *p* = .045), with grit as a full mediator, and depression had a negative effect on self-esteem (H7: β = -0.11, *p* = .007), with grit as a partial mediator (Table 5).

**Table 5:** Hypothesis testing according to family economic status (FES) groups.

Groups	Hypothesized path		Path coefficient	Stdev	t (*p*)	BCa 95% CI	Decision
**Low-FES**	**Direct effects**						
	**H1**	**Social withdrawal → Grit**	-0.23	0.08	2.83 (.005)	[-0.39, -0.07]	Supported
	**H2**	**Social withdrawal → Self-esteem**	0.03	0.07	0.40 (.692)	[-0.11, 0.17]	Not supported
	**H3**	**Depression → Grit**	-0.49	0.07	6.87 (< .001)	[-0.60, -0.32]	Supported
	**H4**	**Depression → Self-esteem**	-0.59	0.06	10.38 (< .001)	[-0.70, -0.48]	Supported
	**H5**	**Grit → Self-esteem**	0.22	0.07	3.09 (.002)	[0.0 9, 0.36]	Supported
	**Indirect effects**						
	**H6**	**Social withdrawal → Grit → Self-esteem**	-0.05	0.03	2.01 (.045)	[-0.11, -0.01]	Supported
	**H7**	**Depression → Grit → Self-esteem**	-0.11	0.04	2.70 (.007)	[-0.20, -0.05]	Supported
**Above-average FES**	**Direct effects**						
	**H1**	**Social withdrawal → Grit**	-0.12	0.04	3.30 (.001)	[-0.20, -0.06]	Supported
	**H2**	**Social withdrawal → Self-esteem**	-0.01	0.03	0.26 (.798)	[-0.07, 0.04]	Not supported
	**H3**	**Depression → Grit**	-0.34	0.03	9.81 (< .001)	[-0.40, -0.27]	Supported
	**H4**	**Depression → Self-esteem**	-0.51	0.03	18.00 (< .001)	[-0.57, -0.46]	Supported
	**H5**	**Grit → Self-esteem**	0.25	0.03	8.25 (< .001)	[0.19, 0.31]	Supported
	**Indirect effects**						
	**H6**	**Social withdrawal → Grit → Self-esteem**	-0.03	0.01	2.98 (.003)	[-0.05, -0.01]	Supported
	**H7**	**Depression → Grit → Self-esteem**	-0.09	0.01	6.32 (< .001)	[-0.11, -0.06]	Supported

Social withdrawal (H1: β = -0.12, *p* = .001) in female adolescents from the above-average FES group had a negative effect on grit, but no effect on self-esteem (H2). Depression had negative effects on grit (H3: β = -0.34, *p* < .001) and self-esteem (H4: β = - 0.51, *p* < .001). Grit also had a positive effect on self-esteem (H5: β = 0.25, *p* < .001). Social withdrawal had a negative effect on self-esteem (H6: β = -0.03, *p* = .003) through grit as a full mediator, and depression had a negative effect on self-esteem (H7: β = -0.09, *p* < .001) as a partial mediator of grit (Table 5).

Therefore, social withdrawal in female adolescents from low-FES group and the above-average FES group had no effect on self-esteem, but causal relationships were shown in the relationships between other variables. However, based on the regression coefficients, social withdrawal or depression in female adolescents from the low-FES group had a more negative effect (direct or indirect) on grit and self-esteem than was observed in the above-average FES group (Table 5).

## Discussion

The current study aimed to elucidate the structural relationships among social withdrawal, depression, grit, and self-esteem in girls in the third year of middle school using panel data from the third year (2020) of the KCYPS 2018. The findings are hoped to shed light on the factors affecting the self-esteem of Korean adolescent girls more generally. The following results were derived.

First, social withdrawal did not appear to have a significant effect on self-esteem in female adolescents, unlike the findings of previous research [28, 29], which found that shyness (a sub-concept of social withdrawal) negatively affected self-esteem. A possible explanation for this may be that withdrawn boys with shy and anxious tendencies are more negatively affected than girls and have internalization problems [61]. This interpretation is supported by a previous study finding that although girls tended to be shyer than boys, the effect of high shyness on girls’ self-esteem was not significant [62]. That is, the effect of social withdrawal on self-esteem differs according to gender, which may explain the finding that social withdrawal in female adolescents did not have a direct effect on self-esteem in this study. Additional research is required to elucidate gender differences in the effect of social withdrawal on self-esteem.

Furthermore, in this study, social withdrawal in female adolescents had a significant negative effect on grit. Studies exploring the relationship between adolescent social withdrawal and grit are extremely rare, and the results of this study are similar to those of a previous study [24] that reported that social withdrawal was negatively correlated with grit. Therefore, this study confirmed that social withdrawal in female adolescents had a negative effect on grit. The results of this study also show that grit can be lower in adolescent girls who feel withdrawn from social relationships and have limited social relationships, partially supporting previous studies [21, 27] in which support from peers and close adults strengthened grit. Female adolescents are more strongly influenced by social relationships than male adolescents [19], and this study also emphasizes the importance of social relationships in strengthening female adolescents’ grit.

Second, in this study, depression in adolescent girls had significant negative effects on grit and self-esteem. This is consistent with the results of a previous study of 18- to 24-year-old college students [13] showing that depression had a negative relationship with grit and self-esteem, and that depression was the strongest factor influencing self-esteem among middle- and high-school adolescents [14]. Female adolescents tend to be more affected by negative emotions such as depression than male adolescents [9], and this study confirmed the negative effects of depression on grit and self-esteem in adolescent girls, suggesting that the management of depression in adolescent girls is very important.

Third, in this study, grit had a significant positive effect on self-esteem in female adolescents. This is consistent with the results of a previous study [22] showing that grit had a significant effect on self-esteem in male and female adolescents. Moreover, other studies have shown that high grit had a positive effect on self-satisfaction [33], academic self-efficacy [23, 34], and self-worth evaluation [32]. Hence, this study reconfirmed that grit contributes to the improvement of self-concept, including self-esteem. In particular, since female adolescents are at higher risk of low self-esteem than male adolescents [9, 10], strategies should be developed to promote self-esteem by improving grit in female adolescents.

Fourth, grit played a complete mediating role in the relationship between social withdrawal and self-esteem of female adolescents. This is partially consistent with the results of a previous study [37] showing a partial mediating effect of grit on the negative relationship between elementary school students’ self-esteem and social withdrawal. In this study, no direct relationship was found between social withdrawal and self-esteem; instead, only an indirect relationship mediated by grit was confirmed, emphasizing the role of grit in the relationship between social withdrawal and self-esteem in female adolescents. In particular, since the tendency for social withdrawal remains relatively stable regardless of time and situation [19], when approaching young girls’ self-esteem, a strategy is needed to improve self-esteem by mitigating the negative effects of social atrophy rather than a direct approach to social withdrawal. A strategy is needed to improve self-esteem by alleviating the negative effects of social withdrawal by improving grit in young girls with social withdrawal.

Fifth, grit played a partial mediating role, attenuating the negative influence of depression on self-esteem in female adolescents in this study. This is similar to the results of existing research on the mediating role of grit in the relationship between depression and negative personality traits [13] and a study finding that grit played a partial mediating role in the relationship between depression and self-resilience [36]. Thus, this study supports the results of previous research suggesting that grit can buffer the negative effects of negative emotions such as depression on self-esteem. In particular, this study confirmed that a grit improvement strategy could be effective for improving low self-esteem due to high depressive tendencies because female adolescents have a higher tendency for depression and a higher risk of low self-esteem than male adolescents.

Sixth, in this study, the strength of relationships between contributing factors and self-esteem in adolescent girls varied according to FES. The low-FES group had a greater decrease in self-esteem due to an increase in depression and a greater decrease in grit due to an increase in depression and social withdrawal than the above-average FES group. Furthermore, the positive direct and indirect effects of grit on self-esteem were weaker in the low FES group than in the above-average FES group. Similar findings were reported in previous studies according to which family income had a significant relationship with the formation of self-esteem [26], and socioeconomic status of family with emotional stabilities and mental problems significantly contributed on self-esteem [15] in adolescents. To summarize, the findings of this study confirm that FES play a significant role in the relationships of emotional factors and grit with self-esteem in adolescent girls.

## Conclusion and practical implications

This study aimed to elucidate the structural relationships among social withdrawal, depression, grit, and self-esteem in female adolescents using large nationally representative data. Depression and grit had significant direct effects on self-esteem in adolescent girls, and grit played a partial mediating role in the relationship between depression and self-esteem. Depression and social withdrawal also had negative effects on grit in adolescent girls. In addition, social withdrawal in female adolescents did not show a significant direct relationship with self-esteem, and grit played a complete mediating role between social withdrawal and self-esteem. Therefore, this study determined that negative emotional states such as depression or social withdrawal in female adolescents have an unfavorable effect on the formation of grit and self-esteem, and that grit plays a mediating role in the relationships of depression and social withdrawal with self-esteem.

The present study suggests that strategies to strengthen grit and help control depression are required to promote self-esteem in female adolescents. Furthermore, designing and implementing a program to reduce depression and social withdrawal would help enhance grit in adolescent girls. In particular, female adolescents tend to be more immersed in and sensitive to their internal mental state than male adolescents [9]; thus, a careful grit and self-esteem improvement strategy that considers their mental state will be important. Strategies to reduce social withdrawal through reinforcement of social networks and support from family, friends, and teachers of female adolescents may also be helpful. In addition, this study found that self-esteem of female adolescents was affected more negatively by depression and influenced less positively by grit in the low-FES group than in the above-average FES group. Hence, economic status should be considered in the interventional approach to self-esteem of female adolescents.

## Limitations and suggestions for future research

The limitations of this study and suggestions for future research are as follows. First, this study is meaningful in that it presented results using large-scale, nationwide data from Korean middle school students using the cohort panel of the KCYPS 2018. However, the panel data, as in other research studies, only included those who were willing to participate, reducing the data’s representativeness of the entire population. The limited generalizability of this study’s findings to all female adolescents in Korea should be considered when interpreting the results of this study. Second, this study analyzed the relationship between grit and other variables by integrating the two dimensions of grit (consistency of interest and perseverance of effort). However, recent studies have discussed differences in the influence of the two dimensions of grit on other variables. Therefore, follow-up studies could derive richer and more meaningful results by dividing grit into two dimensions and investigating their relationships with social withdrawal, depression, and self-esteem. Third, this study had limitations in revealing the causal relationship between variables because the analysis was performed using only the third year of survey data of the three-year panel data. Therefore, follow-up research should conduct longitudinal analyses using methods such as autoregressive cross lag and a latent growth model to establish causal relationships.

## References

[1] Fennell M, Brosan L. An introduction to improving your self-esteem. 2nd ed. London: Robinson; 2020.

[2] McKay MT, Sumnall HR, Cole J.C, Percy A. elf-esteem and self-efficacy: Associations with alcohol consumption in a sample of adolescents in Northern Ireland. Drugs: Educ Prev Policy. 2012;19: 72–80.

[3] Soto-Sanz V, Piqueras JA, Rodríguez-Marín J, Pérez-Vázquez T, Rodríguez-Jiménez T, Castellví P, et al. Self-esteem and suicidal behaviour in youth: A meta-analysis of longitudinal studies. Psicothema. 2019;31: 246–254. doi: 10.7334/psicothema2018.339 31292038

[4] Kim JYJ, Kim E, Lee I. Influence of self-esteem of middle school students for mental care on academic achievement: Based on the mediation effect of GRIT and academic enthusiasm. Int J Environ Res Public Health. 2021;18(13): 7025. doi: 10.3390/ijerph18137025 34209206 PMC8297377

[5] Sowislo JF, Orth U. Does low self-esteem predict depression and anxiety? A meta-analysis of longitudinal studies. Psychol Bull. 2013;139: 213–240. doi: 10.1037/a0028931 22730921

[6] Steiger AE, Allemand M, Robins RW, Fend HA. Low and decreasing self-esteem during adolescence predict adult depression two decades later. J Pers Soc Psychol. 2014;106: 325–338. doi: 10.1037/a0035133 24467425

[7] Masselink M, Van Roekel E, Oldehinkel AJ. Self-esteem in early adolescence as predictor of depressive symptoms in late adolescence and early adulthood: The mediating role of motivational and social factors. J Youth Adolesc. 2018;47: 932–946. doi: 10.1007/s10964-017-0727-z 28785953 PMC5878202

[8] Orth U, Robins RW. The development of self-esteem. Curr Dir Psychol Sci. 2014;23(5): 381–387.

[9] Gomez-Baya D, Mendoza R, Paino S. Emotional basis of gender differences in adolescent self-esteem. Psicologia. 2016;30: 1–14.

[10] Akdemir D, Çak T, Aslan C, Aydos BS, Nalbant K, Çuhadaroğlu-Çetin F. Predictors of self-esteem in adolescents with a psychiatric referral. Turk J Pediatr. 2016;58: 69–78. doi: 10.24953/turkjped.2016.01.010 27922239

[11] Lyyra N, Thorsteinsson EB, Eriksson C, Madsen KR, Tolvanen A, Löfstedt P, et al. The association between loneliness, mental well-being, and self-esteem among adolescents in four Nordic countries. Int J Environ Res Public Health. 2021; 18(14): 7405. doi: 10.3390/ijerph18147405 34299857 PMC8308002

[12] Maldonado L, Huang Y, Chen R, Kasen S, Cohen P, Chen H. Impact of early adolescent anxiety disorders on self-esteem development from adolescence to young adulthood. J Adolesc Health. 2013;53: 287–292. doi: 10.1016/j.jadohealth.2013.02.025 23648133 PMC3725205

[13] Musumari PM, Tangmunkongvorakul A, Srithanaviboonchai K, Techasrivichien T, Suguimoto S. P., Ono-Kihara M, et al. Grit is associated with lower level of depression and anxiety among university students in Chiang Mai, Thailand: A cross-sectional study. PLoS One. 2018;13(12): e02091. 10.1371/journal.pone.0209121 PMC6294431 30550597

[14] Han SS, Kim KM. Influencing factors on self-esteem in adolescents. JKAN. 2006;36(1): 37–44. doi: 10.4040/jkan.2006.36.1.37 16520562

[15] Veselska Z, Madarasova Geckova A, Gajdosova B, Orosova O, van Dijk JP, Reijneveld SA. Socio-economic differences in self-esteem of adolescents influenced by personality, mental health and social support. Eur J Public Health. 2010;20(6): 647–652. doi: 10.1093/eurpub/ckp210 20034930

[16] Health Insurance Review & Assessment Service. Analysis of treatment status for depression and anxiety disorders in the last 5 years (2017–2021) [Internet]. 2022 June 24 [cited 30 Jan 2023]. Available from: https://www.hira.or.kr/bbsDummy.do?pgmid=HIRAA020041000100&brdScnBltNo=4&brdBltNo=10627&pageIndex=1#none.

[17] World Health Organization. Adolescent mental health [Internet]. 2021 Nov 17 [cited 28 Jan 2023]. Available from: https://www.who.int/news-room/fact-sheets/detail/adolescent-mental-health.

[18] Statistics Korea. Statistical results of causes of death in 2020 [Internet]. 2021 Sep 28 [cited 31 Jan 2023]. Available from: https://kostat.go.kr/portal/korea/kor_nw/1/6/2/index.board?bmode=read&bSeq=&aSeq=403046&pageNo=1&rowNum=10&navCount=10&currPg=&searchInfo=&sTarget=title&sTxt.

[19] Rubin KH, Coplan RJ, Bowker JC. Social withdrawal in childhood. Annu Rev Psychol. 2009;60: 141–171. doi: 10.1146/annurev.psych.60.110707.163642 18851686 PMC3800115

[20] Morese R, Palermo S, Torello C, Sechi F. Social withdrawal and mental health: An interdisciplinary approach. In social isolation-an interdisciplinary view. IntechOpen. 2020;3–10. 10.5772/intechopen.90735

[21] Duckworth A. Grit: The power of passion and perseverance. London: Penguin Random House; 2016.

[22] Sohn HG, Kim EH. The Effect of adolescents’ grit and self-esteem on life satisfaction according to gender: The moderated mediating affect of academic helplessness. Korean J Youth Stud. 2022;29(4): 51–74.

[23] Datu JAD, King RB, Valdez JPM, Eala MSM. Grit is associated with lower depression via meaning in life among Filipino high school students. Youth Soc. 2019;51(6): 865–876.

[24] Song HR. The Moderating effect of grit between social withdrawal and happiness of middle school student in poverty. M.Sc. Thesis, Hanyang University. 2020. Available from: https://repository.hanyang.ac.kr/handle/20.500.11754/152857

[25] Yu Y, Wang F, Wang T. A study on the relationships between adolescent inferiority and grit. ASSEHR. 2019;351: 283–288.

[26] Bannink R, Pearce A, Hope S. Family income and young adolescents’ perceived social position: associations with self-esteem and life satisfaction in the UK Millennium Cohort Study. Arch Dis Child. 2016; 101(10): 917–921. doi: 10.1136/archdischild-2015-309651 26957529 PMC5050283

[27] Lan X. Peer attachment and grit in adolescence and emerging adulthood. Psych J. 2019;8(4): 520–521. doi: 10.1002/pchj.289 31066234

[28] Bober A, Gajewska E, Czaprowska A, Świątek AH, Szcześniak M. Impact of shyness on self-esteem: The mediating effect of self-presentation. Int J Environ Res Public Health. 2021;19(1): 230. doi: 10.3390/ijerph19010230 35010490 PMC8744881

[29] Chan SM. Depressive mood in Chinese early adolescents: Relations with shyness, self-esteem and perceived social support. Asia Pac Psychiatry. 2012;4(4): 233–240.

[30] Tang X, Upadyaya K, Salmela-Aro K. School burnout and psychosocial problems among adolescents: Grit as a resilience factor. J Adolesc. 2021;86: 77–89. doi: 10.1016/j.adolescence.2020.12.002 33360420

[31] Li J, Fang M, Wang W, Sun G, Cheng Z. The influence of grit on life satisfaction: Self-esteem as a mediator. Psychol Belg. 2018;58(1): 51–66. doi: 10.5334/pb.400 30479807 PMC6194520

[32] Kleiman EM, Adams LM, Kashdan TB, Riskind JH. Gratitude and grit indirectly reduce risk of suicidal ideations by enhancing meaning in life: Evidence for a mediated moderation model. J Res Pers. 2013;47: 539–546.

[33] Von Culin KR, Tsukayama E, Duckworth AL. Unpacking grit: Motivational correlates of perseverance and passion for long-term goals. J Posit Psychol. 2014;9(4): 306–312. doi: 10.1080/17439760.2014.898320 31404261 PMC6688745

[34] Jiang L, Zhang S, Li X, Luo F. How grit influences high school students’ academic performance and the mediation effect of academic self-efficacy and cognitive learning strategies. Curr Psychol. 2021. 10.1007/s12144-020-01306-x

[35] Credé M, Tynan MC, Harms PD. Much ado about grit: A meta-analytic synthesis of the grit literature. J Pers Soc Psychol. 2017;113(3): 492–511. doi: 10.1037/pspp0000102 27845531

[36] Lee HS, Seo EH. The mediating effects of grit and self-efficacy between depression and ego-resilience of college students. Korean Journal of Educational Research. 2018;56(3): 61–88.

[37] Koo BH, Lee HW, Kim HJ. The structural relations among the parent-child’s self-esteem, Grit, and social withdrawal of children in childhood: Applying the actor-partner interdependence model. Korean J Youth Stud. 2021;28(5): 191–216.

[38] National Youth Policy Institute. User guide of Korean children and youth panel survey 2018. Sejong: National Youth Policy Institute; 2022.

[39] Kim SH, Kim KY. Development of behavior problem scale for children and adolescence. Journal of Families and Better Life. 1998;16: 155–166.

[40] Kim GI, Kim JH, Won HT. Korean manual of symptom checklist-90-revision. Seoul: JoongAng Jeokseong Publisher; 1984.

[41] Kim HM, Hwang MH. Validation of Korean grit scale for children. J Educ. 2015;35: 63–74.

[42] Hewstone M, (Editor), Stroebe Wolfgang. An introduction to social psychology. 7th ed. London: Wiley; 2016.

[43] Curran PJ, West SG, Finch JF. The robustness of test statistics to nonnormality and specification error in confirmatory factor analysis. Psychol Methods. 1996;1(1): 16–29.

[44] Gunkwon Shin, 2018, Partial least squares structural equation modeling(PLS-SEM) with SmartPLS 3.0, Seoul: Chenglam.

[45] Cronbach LJ. Coefficient alpha and the internal structure of tests. Psychometrika. 1951;16: 297–334.

[46] Nunnally J, Bernstein I. Psychometric theory, 3rd ed. New York: MacGraw-Hill: 1994.

[47] Dijkstra TK, Henseler J. Consistent partial least squares path modeling. MIS Quarterly 2015;39(2): 297–316.

[48] Werts CE, Linn RL, Jöreskog KG. Intraclass reliability estimates: Testing structural assumptions. Educ Psychol Meas. 1974;34(1): 25–33.

[49] Bagozzi RP, Yi Y, Phillips LW. Assessing construct validity in organizational research. Adm Sci Q. 1991;36: 421–458.

[50] Hair JF, Ringle CM, Sarstedt M. PLS-SEM: Indeed a silver bullet. J Mark Theory Pract. 2011;19(2): 139–152.

[51] Chin WW. The partial least squares approach to structural equation modeling. Modern Methods for Business Research. 1998;295: 295–336.

[52] Fornell C, Larcker DF. Evaluating structural equation models with unobservable variables and measurement error. J Mark Res. 1981;18(1): 39–50.

[53] Hu LT, Bentler PM. Fit indices in covariance structure modeling: Sensitivity to under parameterized model misspecification. Psychol Methods. 1998;3(4): 424–453.

[54] Bentler PM, Bonett DG. Significance tests and goodness of fit in the analysis of covariance structures. Psychol Bull. 1980;88(3): 588–600.

[55] Wong KKK. Partial least squares structural equation modeling (PLS-SEM) techniques using SmartPLS. Mark bull. 2013;24(1): 1–32.

[56] Hair JF Jr, Sarstedt M, Ringle CM, Gudergan SP. Advanced issues in partial least squares structural equation modeling. CA: SAGE publications; 2017.

[57] Cohen J. Statistical power analysis for the behavioral sciences, 2nd ed. New York: Routledge; 2013.

[58] Fornell C, Cha J. Partial least squares. In advanced methods of marketing research: Bagozzi R., Ed. Cambridge: Blackwell Business; 1994.

[59] Stone M. Cross‐validatory choice and assessment of statistical predictions. J R Stat Soc B: Stat Methodol. 1974;36(2): 111–133.

[60] Geisser S. A predictive approach to the random effect model. Biometrika. 1974;61: 101–107.

[61] Rubin KH, Barstead MG. Gender differences in child and adolescent social withdrawal: A commentary. Sex Roles. 2014;70(7–8): 274–284. doi: 10.1007/s11199-014-0357-9 25709144 PMC4335803

[62] Doey L, Coplan RJ, Kingsbury M. Bashful boys and coy girls: A review of gender differences in childhood Shyness. Sex Roles. 2014;70: 255–266.

